# Selective Cytotoxicity of Single and Dual Anti-CD19 and Anti-CD138 Chimeric Antigen Receptor-Natural Killer Cells against Hematologic Malignancies

**DOI:** 10.1155/2021/5562630

**Published:** 2021-07-11

**Authors:** Sudjit Luanpitpong, Jirarat Poohadsuan, Phatchanat Klaihmon, Surapol Issaragrisil

**Affiliations:** ^1^Siriraj Center of Excellence for Stem Cell Research, Faculty of Medicine Siriraj Hospital, Mahidol University, Bangkok, Thailand; ^2^Division of Hematology, Department of Medicine, Faculty of Medicine Siriraj Hospital, Mahidol University, Bangkok, Thailand; ^3^Bangkok Hematology Center, Wattanosoth Hospital, BDMS Center of Excellence for Cancer, Bangkok, Thailand

## Abstract

Natural killer (NK) cells are part of the first line of defense that rapidly respond to malignant transformed cells. Chimeric antigen receptor- (CAR-) engineered NK cells, although are still at the preliminary stage, have been shown to be alternative to CAR-T cells, mainly due to the absence of graft-versus-host disease and safer clinical profile. Allogeneic human NK cell line NK-92 cells, equipped by CAR, are being developed for clinical applications. Herein, we designed third-generation CARs, optimized the production protocol, and generated CAR-NK-92 cells, targeting CD19 and/or CD138 antigens that employ CD28, 4-1BB, and CD3*ζ* signaling, with >80% CAR expression, designated as CD19-NK-92, CD138-NK-92, and dual-NK-92 cells. The generated CAR-NK-92 cells displayed high and selective cytotoxicity toward various corresponding leukemia, lymphoma, and multiple myeloma cell lines in vitro. Multitargeting approach using a mixture of CD19-NK-92 and CD138-NK-92 cells was also evaluated at various ratios to test the idea of personalized formulation to match the patients' antigen expression profile. Our data indicate that increasing the ratio of CD19-NK-92 to CD138-NK-92 could improve NK cytotoxicity in leukemia cells with a relatively higher expression of CD19 over CD138, supporting the personalized proof of concept. This information represents the basis for further in vivo studies and future progress to clinical trials.

## 1. Introduction

Chimeric antigen receptor- (CAR-) engineered T cell therapy represents a major advancement in personalized cancer therapy, providing hope to patients with relapsed and refractory diseases [1, 2]. Currently, there are three CAR-T cell platforms targeting CD19 approved by the US Food and Drug Administration (FDA) for the treatment of relapsed or refractory acute lymphoblastic leukemia (ALL) in pediatric and young adults (Kymriah®), for the treatment of relapsed or refractory diffused large B-cell lymphoma (DLBCL) in adults (Kymriah® and Yescarta®), and for the treatment of relapsed or refractory mantle cell lymphoma (MCL) in adults (Tecartus™). However, one of the limitations of CAR-T cell therapy is the manufacturing of autologous CAR-T cells from patients, which is laborious, increasing the risk of production failure in clinical settings, especially from those with limited number of healthy T cells, and rendering these CAR-T cells unsuitable for patients with rapidly progressing disease. The manufacturing process could take up to 4 weeks with additional 2-week treatment-free period prior to apheresis to ensure sufficient cell numbers and viability [3].

Natural killer (NK) cells are part of the first line of defense that are rapidly activated to protect body against foreign materials and abnormal cells, including malignant transformed cells, without prior sensitization, representing an important effector cell type for cellular immunotherapy. Increasing evidence has shown that allogeneic NK cells have stronger tumor killing ability than autologous NK cells and that they do not carry the risk of inducing graft-versus-host disease (GvHD) frequently associated with allogeneic T cells [4]. However, primary NK cells have a limited life span and proliferative capacity that restrict their clinical applications. Human NK cell line NK-92 cells, which exhibit a high degree of cytotoxicity toward various cancer cells, have become the major allogeneic source of NK cells and are the only NK cell line approved by the US FDA for phase I and II clinical trials. NK-92 cells can be continuously expanded in the presence of interleukin-2 (IL-2) in a current good manufacturing practice- (cGMP-) compliant process [5].

The natural antitumor properties, ability to cultivate, and early-phase clinical safety profile of NK-92 cells make them a promising cell source for the implementation of CAR to establish off-the-shelf, universal CAR-NK cells, which have the basic framework of CAR-T cells. In comparison to CAR-T cells, a far fewer preclinical and clinical studies have been performed for CAR-NK cells, most of which aim to target and treat solid tumors. The feasibility of generation of first- and second-generation CAR-NK-92 cells targeting CD19 has been demonstrated thus far. In addition to CD19 antigen, which expresses commonly in multitude of B-cell malignancies and likely in stem cell-like subpopulation of multiple myeloma (MM) [6], CD138 (syndecan-1) has gained our attention. CD138, a member of the syndecan family involved in cell-cell and cell-matrix interaction, is a known marker of MM associated with its growth and cell proliferation [7]. The expression of CD138 has also been detected in lymphoplasmacytic lymphoma (LPL), B-cell chronic lymphocytic leukemia (B-CLL), and certain cases of acute lymphoblastic leukemia (ALL) and acute myeloblastic leukemia (AML) [8].

In the present study, we investigated the feasibility of generating third-generation CAR-NK-92 cells targeting CD19 and CD138 as well as dual CAR-NK-92 cells cotargeting CD19 and CD138 and evaluated their selective cytotoxicity in vitro toward various hematologic cancer cells with different CD19 and CD138 expression profiles. We also evaluated the cytotoxic effects of the mixture of CAR-NK-92 cells targeting CD19 and CD138 to test the idea of personalized formulation to match the patients' antigen expression profile.

## 2. Materials and Methods

### 2.1. Cell Culture

Human embryonic kidney (HEK) 293T cells, human NK-92 cells, and hematologic cancer cell lines, including human chronic myeloid leukemia- (CML-) derived K562 cells [9], human ALL-derived REH cells [10], human Burkitt's lymphoma- (BL-) derived Raji cells [11], and human MM-derived H929 and RPMI-8226 [12] were obtained from American Type Culture Collection (ATCC, Manassas, VA). Mycoplasma contamination was checked every eight weeks using MycoAlert mycoplasma detection kit (Lonza, Cologne, Germany), and any cell lines found positive were discarded. NK-92 cells were cultured in an Alpha Minimum Essential Medium (MEM-*α*; Gibco brand, Thermo Fisher Scientific, Waltham, MA) containing 100 U/mL recombinant human (rh) IL-2 (Miltenyi Biotec, Bergisch Gladbach, Germany), 12.5% horse serum (Invitrogen, Carlsbad, CA), and 12.5% fetal bovine serum (FBS; Merck Millipore, Darmstadt, Germany). HEK293T cells were cultured in Dulbecco's Modified Eagle's Medium (DMEM), while all other cells were cultured in RPMI 1640 medium containing 10% FBS, 100 U/mL penicillin, and 100 *μ*g/mL streptomycin. All cells were maintained in a humidified atmosphere of 5% CO_2_ environment at 37°C.

### 2.2. Construction of CAR Vectors

**Figure 1:**
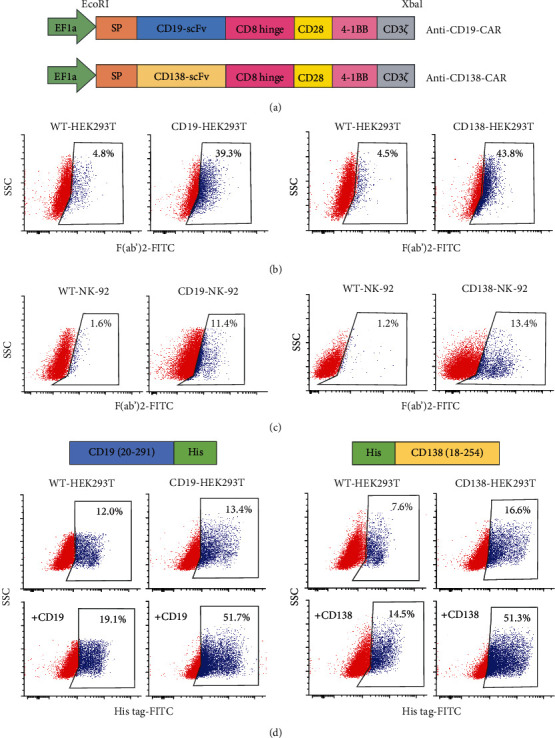
Construction of anti-CD19-CAR and anti-CD138-CAR and detection of CAR expression. (a) Diagram of the lentiviral vectors encoding third-generation CARs to target CD19 (*upper*) or CD138 (*lower*) antigen, designated as anti-CD19-CAR or anti-CD138-CAR, under the control of EF1a promoter and the restriction enzyme cutting sites EcoRI and Xbal. (b) CAR expression on the surface of CD19-HEK293T (*left*) or CD138-HEK293T (*right*) cells after transfection of anti-CD19-CAR or anti-CD138-CAR together with pCMV.dR8.2 dvpr and pCMV-VSV-G plasmids, as evaluated by flow cytometry using anti-F(ab′)2 antibody. (c) CAR expression in CD19-NK-92 (*left*) or CD138-NK-92 (*right*) cells after first round of lentiviral transduction, as similarly evaluated using anti-F(ab′)2 antibody. (d) CAR expression in CD19-HEK293T (*left*) or CD138-HEK293T (*right*) cells, as evaluated by flow cytometry based on its antigen-binding activity to His tag-rhCD19 or His tag-rhCD138.

Third-generation CARs targeting CD19 and CD138 with CD3*ζ* signaling domain and costimulatory domains CD28 and 4-1BB, designated as anti-CD19 CAR and anti-CD138 CAR, respectively, were constructed by Creative Biolabs (Shirley, NY). Specifically, the full length of signal peptide, anti-CD19 scFv from FMC63, and anti-CD138 scFV from indatuximab with CD8 hinge, CD28, 4-1BB, and CD3*ζ* were synthesized and subcloned into lentiviral vector Lenti-EF1a-AT-Free. The inserts were confirmed by Sanger sequencing, and the structures of CAR vectors are schematically illustrated in Figure 1(a).

### 2.3. Lentiviral Production

Lentiviral production was performed using HEK293T packaging cells in conjunction with pCMV.dR8.2 dvpr lentiviral packaging and pCMV-VSV-G envelope plasmids (Addgene #8454 and #8455) [13]. Briefly, HEK293T cells were transfected with anti-CD19 CAR or anti-CD138 CAR, pCMV.dR8.2 dvpr, and pCMV-VSV-G plasmids at the ratio of 12 : 5: 1 using Lipofectamine 3000 (Thermo Fisher Scientific) and were checked for the CAR expression by flow cytometry at 48 h posttransfection to ascertain the mammalian expression of the constructed CAR vectors. The lentiviral particles were harvested and pooled at approximately 24 and 48 h posttransfection and were concentrated using Amicon Ultra-15 centrifugal filters (Merck Millipore, Tullagreen, Ireland).

### 2.4. Evaluation of CAR Expression by Flow Cytometry

CAR expression was determined by flow cytometry based on Fab fragments as well as target antigens. For anti-Fab staining, cells were incubated with FITC- (fluorescein-) conjugated anti-mouse-IgG, F(ab′)2 fragment antibody (F(ab) ′2-FITC; Jackson ImmunoResearch, West Grove, PA) for 30 min at 4°C and analyzed using a FACScalibur flow cytometer (BD Biosciences, San Jose, CA USA).

For target antigen-based detection, cells were incubated with 10 *μ*g/mL rh CD19 (20-291) protein with His tag to the C-terminus (Abcam, Cambridge, UK) or rh CD138 (18-254) protein with His tag to the N terminus (Abcam) for 1 h at 4°C, followed by an incubation with FITC-conjugated anti-His tag antibody (His tag-FITC; Abcam) for 15 min at room temperature and flow cytometric analysis. Cells that were incubated with His tag-FITC, but not with rh CD19 or rh CD138, were used as a basal control. The percentage of cells that expressed CAR could be calculated from the subtraction of FITC-positive cells in basal control from those with target proteins.

### 2.5. Generation of Anti-CD19 and Anti-CD138 CAR-NK-92 Cells

NK-92 cells were transduced with lentiviral particles using spinoculation method. Briefly, low-volume NK-92 cells at the density of 5 × 10^5^ cells/200 *μ*L were incubated with the concentrated viral particles in the presence of Vectofusin-1 (10 *μ*g/mL) (Miltenyi Biotec), followed by a spinoculation at 400 × g for 2 h at room temperature. After which, media containing Vectofusin-1 were added and incubated for additional 24 h before they were removed and replaced with fresh medium. NK-92 cells were evaluated for CAR expression by Fab detection as described above at 72 h posttransduction, and F(ab′)2-FITC-positive cells were enriched by fluorescence-activated cell sorting (FACS) using FACSAria cell sorter (BD Biosciences). Cell transduction and sorting were repeated thrice to ensure that more than 80% of the cells in culture were positive for F(ab′)2-FITC, an indication of anti-CD19 or anti-CD138 scFv expression. The enriched anti-CD19 CAR-NK-92 and anti-CD138 CAR-NK-92 cells, designated as CD19-NK-92 and CD138-NK-92 cells, respectively, were reevaluated for CD19-CAR or CD138-CAR expression by target antigens.

### 2.6. Generation of Dual Targeted Anti-CD19 and Anti-CD138 CAR-NK-92 Cells

Stable, enriched CD138-NK-92 cells were transduced with lentiviral particles containing CD19-CAR in the presence of Vectofusin-1 (10 *μ*g/mL) using spinoculation method as described above. Cells were evaluated for CAR expression based on CD19 target antigen at 72 h posttransduction, and His tag-FITC-positive cells (anti-CD19 CAR-positive cells) were enriched by FACS using FACSAria cell sorter. Cell transduction and sorting were repeated twice, and the enriched dual anti-CD19 and anti-CD138 CAR-NK-92 (dual-NK-92) cells were verified for CD138-CAR expression by CD138 target antigen.

### 2.7. CD19 and CD138 Surface Marker Analysis

Target cancer cells were analyzed for CD19 and CD138 surface expression by incubating with FITC-conjugated anti-human CD19 and PE-conjugated CD138 antibodies for 15 min at room temperature and analyzed using the FACScalibur flow cytometer.

### 2.8. NK Cell Cytotoxicity Assay

Target cancer cells were labeled with PKH67 green fluorescent lipophilic dye for 10 min at 37°C and were mixed with the corresponding ratio of CD19-, CD138-, or dual CD19/CD138-CAR-NK-92 cells for various times (0–24 h). After which, target cell death was evaluated by Annexin V/7-AAD assay. The mixture of effector NK-92 cells and target cancer cells was harvested, washed, and stained with PE-conjugated Annexin V and 7-AAD in binding buffer supplemented with 5 mmol/L calcium chloride for 15 min at room temperature. Samples were immediately analyzed by the FACScalibur flow cytometer with a specific gating strategy to identify the percentage of Annexin V single-positive cells defining early apoptosis, Annexin V and 7-AAD double-positive cells defining late apoptosis/necrosis, and 7-AAD single-positive cells defining necrosis, in the PKH67-positive subpopulation. Total cell death of target cancer cells was Annexin V- and/or 7-AAD-positive cells in the PKH67-positive subpopulation.

### 2.9. PKH67/Hoechst 33342 Double Staining and Apoptosis Detection

Target cancer cells were labeled with PKH67 dye for 10 min at 37°C and were mixed with the corresponding ratio of CD19- or CD138-CAR-NK-92 cells for 4 h. The coculture of NK-92 cells and target cancer cells was incubated with 10 mg/mL Hoechst 33342 (Molecular Probes, Eugene, Oregon, USA) for 30 min, after which they were visualized for green florescence under a florescence microscope (Eclipse Ti-U with NiS-Elements, Nikon) and marked for PKH67-positive (target) cells. Apoptosis of PKH67-positive cells was analyzed under blue florescence by scoring the percentage of cells having condensed chromatin and/or fragmented nuclei. The apoptotic index was calculated as the percentage of cells with apoptotic nuclei over the total number of PKH67-positive cells.

### 2.10. Statistical Analysis

The data represent means ± s.d. from three or more independent experiments. Statistical analysis was performed by two-sided Student's *t*-test or one-way ANOVA followed by a Bonferroni posttest at a significance level of *p* < 0.05.

## 3. Results

### 3.1. Construction of Anti-CD19 CAR and Anti-CD138 CAR and Validation for CAR Expression in HEK293T Cells

We constructed the third-generation CARs to target CD19 and CD138 antigens, which both harbor an anti-CD19 or anti-CD139 scFv fragment consisting of heavy and light chains, linked to human CD28, 4-1BB, and CD3*ζ* signaling domains via a CD8 hinge region with a signal peptide sequence (MALPVTALLLPLALLLHAARP) (Figure 1(a)). Notably, these CAR designs and constructs can also be used to generate anti-CD19 or anti-CD138 CAR-T cells. VSV-G-pseudotyped lentiviral vector particles were produced using HEK293T cells and used for transduction of NK-92 cells. Due to the relatively large size of CAR vectors (~9 kb) and the limited efficiency of gene transfer into NK cells when compared with other human cells, including T cells, we first evaluated the CAR expression in anti-CD19- and anti-CD138-transfected HEK293T cells, designated as CD19-HEK293T and CD138-HEK293T cells, in comparison to their wild-type (WT) counterpart. The results show that scFv expression on the surface of CD19-HEK293T and CD138-HEK293T cells, as evaluated by flow cytometry using anti-F(ab′)2 antibody, was approximately 40% for both CARs (Figure 1(b)) and that the produced lentiviral particles from these cells were able to transduce NK-92 cells into CD19-NK-92 and CD138-NK-92 cells with the transduction efficiency of approximately 10% (Figure 1(c)). Alternatively, the specific anti-CD19 and anti-CD138 CAR expression, as evaluated by their binding activities to corresponding antigens fused with His tag, yielded similar results to those of anti-F(ab′)2 staining (Figure 1(d)), thus confirming the practical CAR constructs and the reliable detection methods.

### 3.2. FACS Enrichment of CD19-NK-92 and CD138-NK-92 Cells

**Figure 2:**
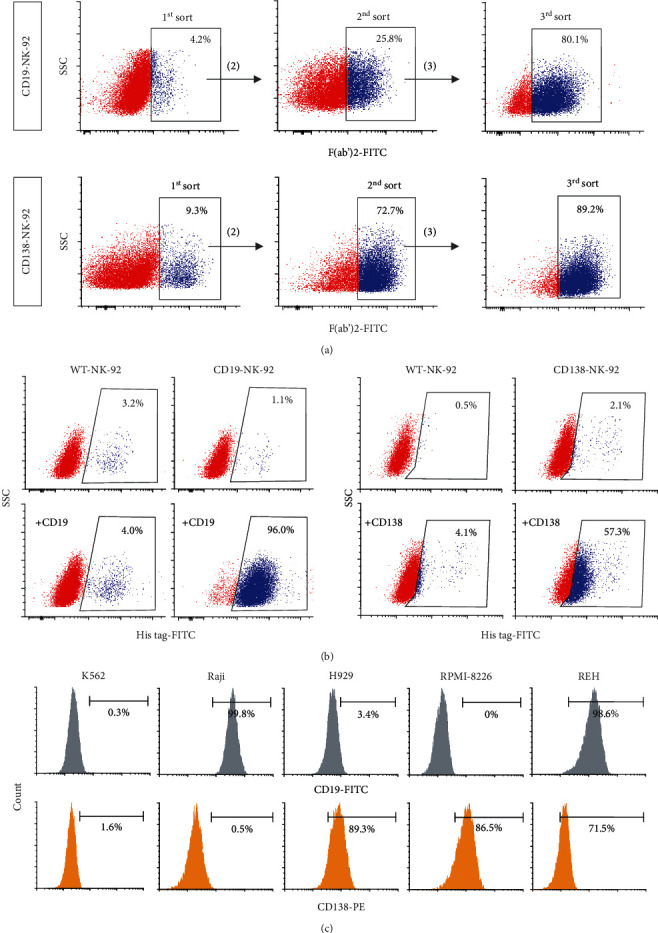
Generation of CD19-NK-92 and CD138-NK-92 cells and analysis of surface antigens in target cancer cells. (a) NK-92 cells were subjected to multiple rounds of lentiviral transduction of anti-CD19-CAR or anti-CD138-CAR and sequential FACS enrichment using anti-F(ab′)2 antibody to generate highly expressed, stable CD19-NK-92 and CD138-NK-92 cells. (b) CAR expression in enriched CD19-NK-92 (*left*) and CD138-NK-92 (*right*) cells, as evaluated by flow cytometry based on its binding activity to His tag-rhCD19 or His tag-rhCD138. (c) Flow cytometric analysis of surface CD19 and CD138 expression in human CML-derived K562, human ALL-derived REH, human BL-derived Raji, and human MM-derived H929 and RPMI-8226 cells.

With the relatively low transduction efficiency in NK-92 cells, anti-CD19- and anti-CD138-transduced NK-92 cells were generated by three rounds of lentiviral transduction and subsequent enrichment by FACS sorting, resulting in homogeneous (>80%) F(ab′)2-positive CD19-NK-92 and CD138-NK-92 cells (Figure 2(a)). The enriched CD19-NK-92 and CD138-NK-92 cells were confirmed to exhibit corresponding CD19 and CD138 binding activities at approximately 90% and 50%, respectively (Figure 2(b)).

### 3.3. Expression of CD19 and CD138 Antigens in Various Hematologic Cancer Cells

To test the cytotoxicity and specificity of established CD19-NK-92 and CD138-NK-92 cells, we first assessed the expression of CD19 and CD138 surface antigens in various hematologic cancer cell lines to identify representative target cells with differential CD19 and CD138 expression profiles.  Figure 2(c) shows that while the majority (>98%) of human CML-derived K562 cells were negative for CD19 and CD138, human ALL-derived REH cells were shown to be positive for both antigens (>98% CD19; >70% CD138). Human BL-derived Raji cells were identified as CD19-positive cells alone (>99% CD19; <1% CD138), and human MM-derived H929 and RPMI-8226 cells were identified as CD138-positive cells alone (>85% CD138; <4% CD19) herein.

### 3.4. CD19-NK-92 and CD138-NK-92 Cells Show Dose- and Time-Dependent Cytotoxicity against Target Cells

**Figure 3:**
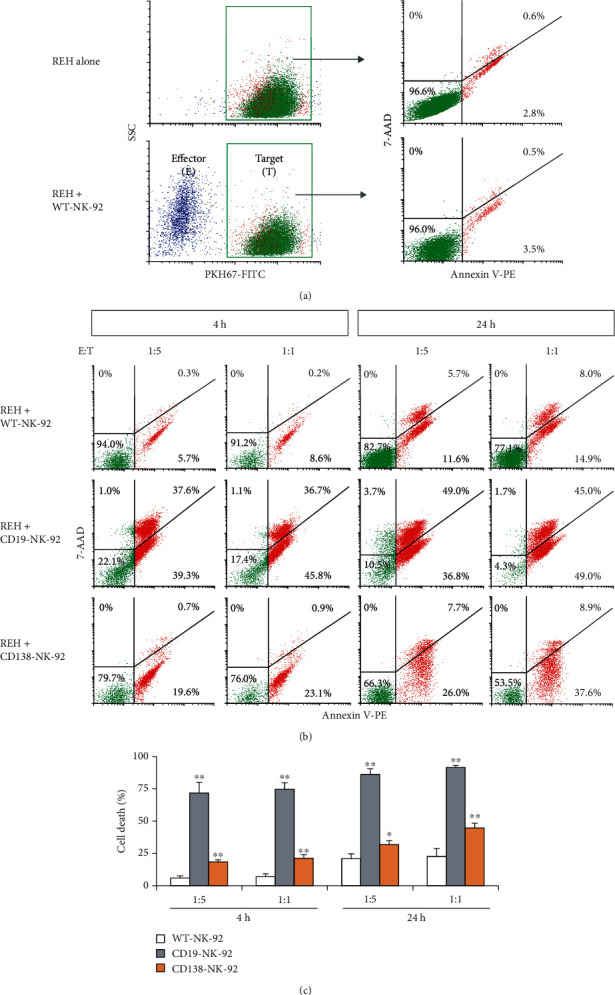
Dose and time profiles of the cytotoxicity of CD19-NK-92 and CD138-NK-92 cells toward ALL cells expressing both CD19 and CD138 antigens. (a) Flow cytometry gating strategy to specifically detect cell death of PKH67-labeled target (T) cells, i.e., human ALL-derived REH cells, after incubation with effector NK (E) cells, i.e., WT-NK-92 cells, by Annexin V/7-AAD assay. (b) PKH67-labeled REH cells were incubated with WT-NK-92, CD19-NK-92, or CD138-NK-92 cells at the *E* :  *T* ratio of 1 : 5 or 1 : 1 for 4–24 h, after which they were stained with Annexin V-PE and 7-AAD. Representative dot plots of Annexin V-PE versus 7-AAD in PKH67-labeled REH cells were shown. (c) Percentage of total REH cell death, comprising Annexin V- and/or 7-AAD-positive cells, was plotted. Data are mean ± s.d. (*n* = 3). ^∗^*p* < 0.05 and ^∗∗^*p* < 0.01 versus WT-NK-92 cells in the same condition; two-sided Student's *t*-test.

To initially evaluate the cytotoxicity of CD19-NK-92 and CD138-NK-92 cells, human ALL-derived REH cells, which expressed both CD19 and CD138 surface antigens, were chosen. REH cells, so-called target cells (*T*), were labeled with PKH67 green fluorescence and cocultured with CD19-NK-92, CD138-NK-92, or WT-NK-92 cells, so-called effector cells (*E*), with an *E* : *T* ratio of 1 : 5 or 1 : 1 for 4–24 h. Cell death rate of PKH67-positive target cells was then evaluated by Annexin V/7-AAD assay using the gating strategy shown in Figure 3(a). The results show that REH cells were considerably resistant to killing by parental WT-NK-92 cells at 4 h though the death rate increased with time and dose of WT-NK-92 cells. CD19-NK-92 cells were found to be the most effective in killing REH cells in a dose- and time-dependent manner, with the death rate at the *E* : *T* ratio of 1 : 5 and 1 : 1 of approximately 70% and 80% at 4 h and 80% and 90% at 24 h, respectively, versus 20% and 25% at 4 h and 30% and 35% at 24 h by CD138-NK-92 cells (Figures 3(b) and 3(c)). Notably, the lesser extent in killing by CD138-NK-92 cells was in line with the lower expression of CD138 when compared with CD19 in REH cells.

### 3.5. Selective Cytotoxicity of CD19-NK-92 and CD138-NK-92 Cells against Various Hematologic Cancer Cells

**Figure 4:**
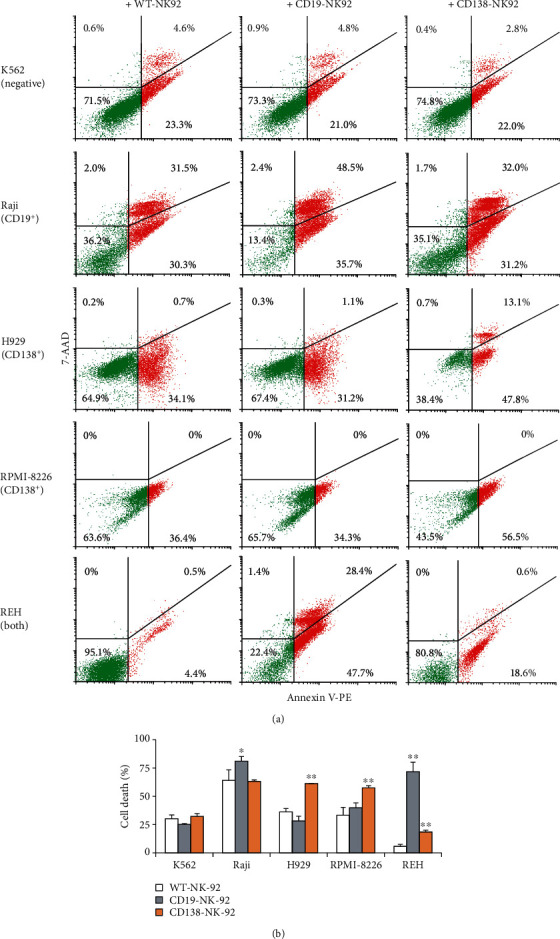
Selective cytotoxicity of CD19-NK-92 and CD138-NK-92 cells toward various hematologic cancer cells. (a) Human CML-derived K562, BL-derived Raji, MM-derived H929 and RPMI-8226 cells, and ALL-derived REH were labeled with PKH67 dye; incubated with WT-NK-92, CD19-NK-92, or CD138-NK-92 cells at the *E* : *T* ratio of 1 : 5 for 4 h; and stained with Annexin V-PE and 7-AAD. Representative dot plots of Annexin V-PE versus 7-AAD in PKH67-labeled cells were shown. (b) Percentage of total cancer cell death was plotted. Data are mean ± s.d. (*n* = 3). ^∗^*p* < 0.05 and ^∗∗^*p* < 0.01 versus WT-NK-92 cells; two-sided Student's *t*-test.

To further determine the specificity of cytotoxicity of CD19-NK-92 cells toward CD19 antigen and CD138-NK-92 cells toward CD138 antigen, the CD19-positive Raji cells and CE138-positive H929 and RPMI-8226 cells were subjected to NK cell cytotoxicity assay in comparison with CD19- and CD138-negative K562 cells and CD19- and CD138-positive REH cells at the *E* : *T* ratio 1 : 5 at 4 h. Figures 4(a) and 4(b) show that CD19-NK-92 cells significantly improved the NK killing in Raji and REH cells when compared to their respective WT-NK-92 control, while having a minimal effect on K562, H929, and RPMI-8226 cells, indicating its specificity toward target cancer cells presenting CD19 antigen. Likewise, CD138-NK-92 cell killing was evidently higher than that of WT-NK-92 cells only in REH, H929, and RPMI-8226 cells, assuring its specificity toward target cells presenting CD138 antigen. Together, our established CD19-NK-92 and CD138-NK-92 cells were highly specific to their antigens without showing cross-reactivity between CD19 and CD138 and were highly cytotoxic toward various corresponding hematologic cancer cells as both CAR-NK cytotoxicities were observed at a relatively low *E* : *T* ratio of 1 : 5 and at as early as 4 h postincubation.

### 3.6. CD19-NK-92 and CD138-NK-92 Cells Induced Apoptosis of Target Cancer Cells

**Figure 5:**
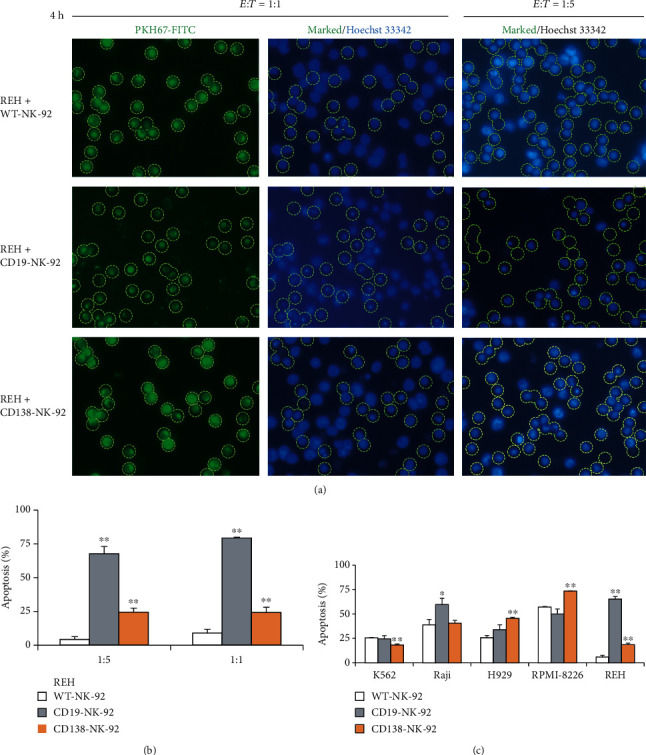
CD19-NK-92 and CD138-NK-92 cells selectively induced apoptosis of target cancer cells. Human ALL-derived REH cells were labeled with PKH67 dye; incubated with WT-NK-92, CD19-NK-92, or CD138-NK-92 cells at the *E* : *T* ratio of 1 : 1 or 1 : 5 for 4 h; stained with Hoechst 33342; and visualized under a fluorescence microscope. (a) Micrographs of PKH67-positive cells under green fluorescence were used to mark the target cells and apoptosis of target cells was scored under blue florescence based on the fragmented/condensed nuclei. (b) Percentage of apoptosis over total REH cells was plotted. Data are mean ± s.d. (*n* = 3). ^∗^*p* < 0.05 and ^∗∗^*p* < 0.01 versus WT-NK-92 cells at the same condition; two-sided Student's *t*-test. (c) Apoptosis of PKH67-labeled CML-derived K562, BL-derived Raji, MM-derived H929 and RPMI-8226, and ALL-derived REH cells upon incubation with WT-NK-92, CD19-NK-92, or CD138-NK-92 cells at the *E* : *T* ratio of 1 : 5 for 4 h was similarly determined by Hoechst 33342 assay. Percentage of apoptosis over total target cells was plotted. Data are means ± s.d. (*n* = 3). ^∗^*p* < 0.05 and ^∗∗^*p* < 0.01 versus WT-NK-92 cells; two-sided Student's *t*-test.

Annexin V/7-AAD assay demonstrated that CD19-NK-92 and CD138-NK-92 cells effectively killed its target cancer cells and suggested that the major mode of cell death was apoptosis. We next used Hoechst 33342 assay to evaluate the apoptosis of human ALL-derived REH cells upon incubation with CD19-NK-92 and CD138-NK-92 cells at the *E* : *T* ratio of 1 : 5 or 1 : 1 for 4 h. REH cells were first labeled with PKH67 green fluorescence, cocultured with CD19-NK-92, CD138-NK-92, or WT-NK-92 cells at the indicated concentrations, and subsequently stained with Hoechst 33342 dye. The apoptosis of PKH67-positive REH cells was scored based on the fragment and/or condensed nuclei and presented as the percentage of apoptosis over total PKH67-positive cells. Figures 5(a) and 5(b) show that CD19-NK-92 cells remarkably induced REH apoptosis in a dose-dependent manner, while CD138-NK-92 cells at the *E* : *T* ratio of 1 : 5 and 1 : 1 yielded similar apoptotic effect, in agreement with the data obtained from Annexin V/7-AAD assay. By comparing the percentage of total cell death and percentage of apoptosis of REH cells at the same condition, we could validate that apoptosis is the major mode of cell death induced by CD19-NK-92 and CD138-NK-92 cells in these experimental settings. The apoptosis rate of CD19-positive Raji cells and CE138-positive H929 and RPMI-8226 cells over CD19-NK-92 and CD138-NK-92 cells further confirmed the selectivity and efficiency of CAR-NK-92 cells (Figure 5(c)).

### 3.7. FACS Enrichment of Dual CD19/CD138-NK-92 Cells and Its Selective Cytotoxicity against Various Hematologic Cancer Cells

**Figure 6:**
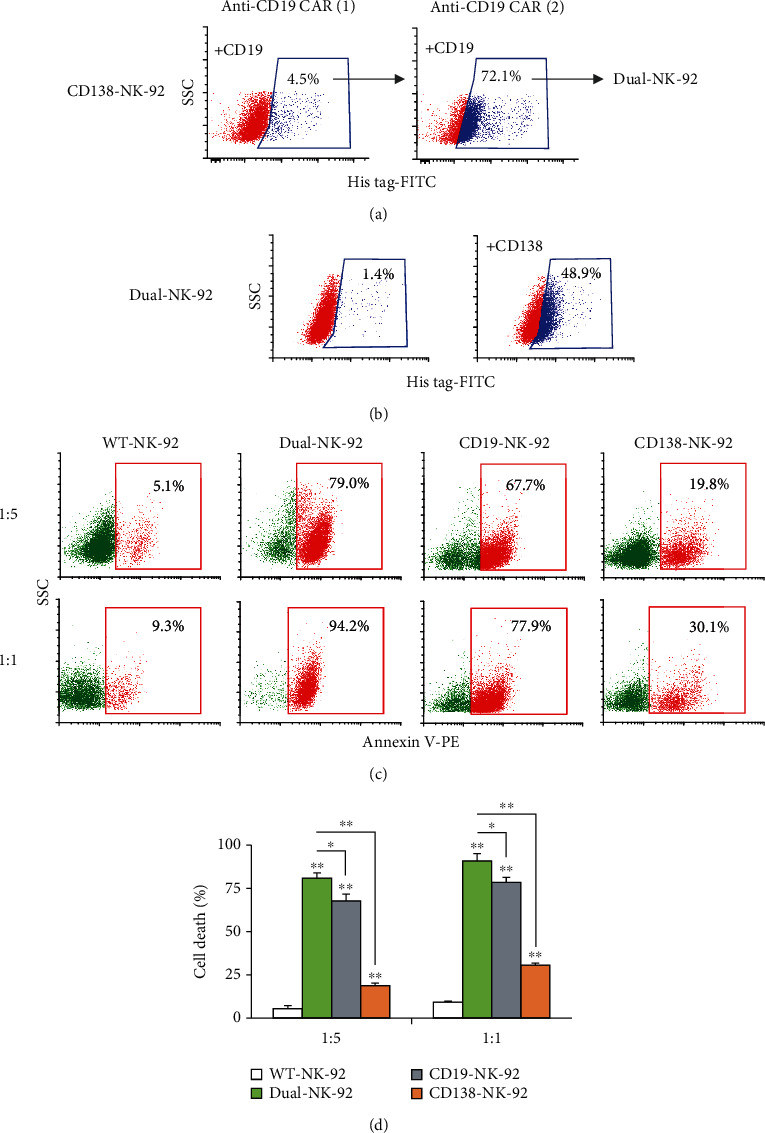
Generation of dual CD19/CD138-NK-92 cells and its cytotoxicity against ALL cells expressing both CD19 and CD138 antigens. (a) Stable CD138-NK-92 cells were subjected to multiple rounds of lentiviral transduction of anti-CD19-CAR and sequential FACS enrichment based on CD19 antigen-binding activity to obtain dual-NK-92 cells. (b) Anti-CD138-CAR expression in dual-NK-92 cells, as evaluated by flow cytometry based on its antigen-binding activity to His tag-rhCD138. (c) Human ALL-derived REH cells were labeled with PKH67 dye; incubated with WT-NK-92, dual-NK-92, CD19-NK-92, or CD138-NK-92 cells at the *E* : *T* ratio of 1 : 5 or 1 : 1 for 4 h; and stained with Annexin V-PE. Representative flow cytometric dot plots of Annexin V-positive cells (*red box*) in PKH67-labeled cells were shown. (d) Percentage of REH cell death was plotted. Data are mean ± s.d. (*n* = 3). ^∗^*p* < 0.05 and ^∗∗^*p* < 0.01 versus WT-NK-92 cells or dual-NK-92 cells as indicated; two-sided Student's *t*-test.

Certain cases of hematologic cancer cells, particularly LPL, B-CLL, ALL, and AML, might coexpress CD19 and CD138 [8]. We therefore generated dual-NK-92 cells cotargeting both CD19 and CD138 by stepwise transduction of anti-CD19 CAR into CD138-NK-92 cells and FACS enrichment based on CD19 antigen detection (Figure 6(a)) and investigated whether it would improve the killing ability against targets with both antigens.  Figure 6(b) shows that the enriched dual-NK-92 cells with >80% CD19-binding activity retained their original CD138-binding activity. The cytotoxicity of dual-NK-92 cells was tested against CD19- and CD138-positive REH cells at the *E* : *T* ratio of 1 : 5 and 1 : 1 at 4 h in comparison to their single CD19-NK-92 and CD138-NK-92 counterparts. The results show that dual-NK-92 cells indeed exhibited superior cell death rate of REH cells over the single-targeted CD19-NK-92 and CD138-NK-92 cells in a dose-dependent fashion (Figures 6(c) and 6(d)), thereby confirming that dual-NK-92 cells can attack dual targets and improve NK cytotoxicity.

**Figure 7:**
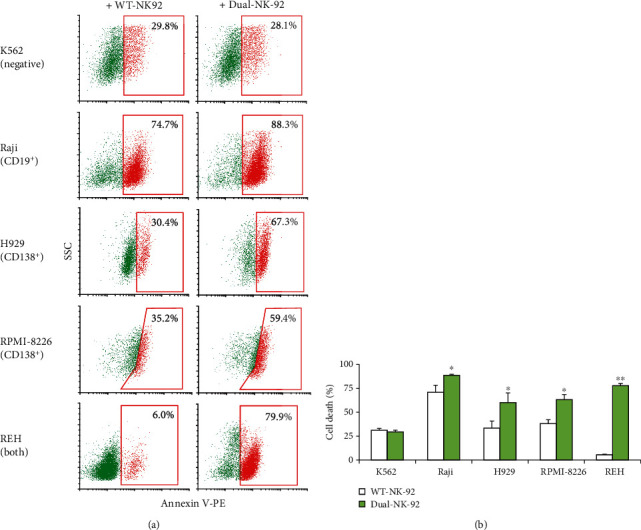
Selective cytotoxicity of dual CD19/CD138-NK-92 cells toward various hematologic cancer cells. (a) Human CML-derived K562, ALL-derived REH, BL-derived Raji, and MM-derived H929 and RPMI-8226 cells were labeled with PKH67 dye, incubated with WT-NK-92 or dual-NK-92 cells at the *E* : *T* ratio of 1 : 5 for 4 h, and stained with Annexin V-PE. Representative flow cytometric dot plots of Annexin V-positive cells (*red box*) in PKH67-labeled cells were shown. (b) Percentage of cancer cell death was plotted. Data are mean ± s.d. (*n* = 3). ^∗^*p* < 0.05 and ^∗∗^*p* < 0.01 versus WT-NK-92 cells; two-sided Student's *t*-test.

To ascertain the specificity of dual-NK-92 cells, we performed its cytotoxicity toward various hematologic cancer cells with different CD19 and CD138 profiles. Figures 7(a) and 7(b) demonstrate the killing capability of dual-NK-92 cells toward all tested cells presenting either of CD19 or CD138 or both, including Raji, H929, RPMI-8226, and REH cells, but not CD19- and CD138-negative K562 cells, and thus validating the selective cytotoxicity of dual-NK-92 cells.

### 3.8. Customized Titration of the Mixture of CD19-NK-92 and CD138-NK-92 Cells

**Figure 8:**
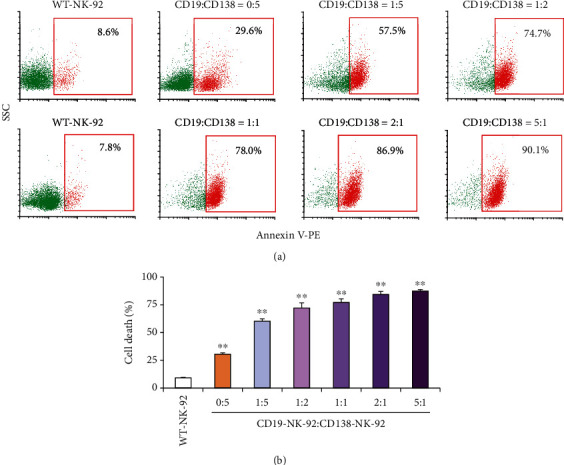
Cytotoxicity of the mixture of CD19-NK-92 and CD138-NK-92 cells toward ALL cells expressing CD19 and, to a lesser extent, CD138 antigens. (a) Human ALL-derived REH cells were labeled with PKH67 dye, incubated with WT-NK-92 cells or the mixture CD19-NK-92 and CD138-NK-92 cells at various ratios (0 : 5˗5 : 1) at the final *E* : *T* ratio of 1 : 1 for 4 h, and stained with Annexin V-PE. Representative flow cytometric dot plots of Annexin V-positive cells (*red box*) in PKH67-labeled cells were shown. (b) Percentage of REH cell death was plotted. Data are mean ± s.d. (*n* = 3). ^∗∗^*p* < 0.01 versus WT-NK-92 cells; two-sided Student's *t*-test.

Variations in surface antigen expression could be observed among patients with hematologic malignancies, even with the same origins or subtypes. To test the idea of personalized CAR-NK-92 cells to match the patients' antigen profile, we evaluated the NK cytotoxicity of the mixture of CD19-NK-92 and CD138-NK-92 cells at various ratios of CD19-NK-92 : CD138-NK-92, e.g., 0 : 5 (CD138-NK-92 cells only), 1 : 5, 1 : 2, 1 : 1, 2 : 1, and 5 : 1, at the fixed *E* : *T* ratio of 1 : 1 at 4 h in CD19- and CD138-positive REH cells. With the higher and stronger expression of CD19 antigen in REH cells, we postulated that an increase in CD19-NK-92 : CD138-NK-92 ratio would lead to a more pronounced killing ability. As expected, there was a remarkable dose-dependent increase in cell death rate by the CAR-NK-92 mixture attributable to the higher proportion of CD19-NK-92 cells (Figure 8), supporting the potential application of the customized CAR-NK-92 mixture that was not limited to targeting CD19 and CD138 in the future.

## 4. Discussion

In the present study, we generated three new third-generation CAR-NK-92 cells, including CD19-NK-92, CD138-NK-92, and dual-NK-92 cells, targeting CD19 and/or CD138 antigens on the surface of lymphomas, leukemias, and/or MM, and analyzed their cytotoxicity and selectivity in various hematologic cancer cells with different CD19 and CD138 profiles. Our established CAR-NK-92 cells displayed high selectivity toward their corresponding tumor antigens with remarkable cytotoxicity. It is worth noting that we observed significant cytotoxic effects of both CD19-NK-92 and CD138-NK-92 cells at the relatively low *E* : *T* ratio of 1 : 5 at 4 h postincubation, while previous reports on the efficacy of first- and second-generation CAR-NK-92 cells targeting CD19 or CD138 in similar hematologic cancer cell lines, albeit with different experimental settings, generally evaluated CAR-NK-92 effects at the *E* : *T* ratio ranging from 1 : 4 at 6–24 h postincubation to 5 : 1 at 4 h postincubation (Supplementary Table S1). In real-life scenario, CAR-NK-92 cells against one or two specific antigens will not suit all patients. The idea of multitargeting approach using a combination of different CAR-NK-92 cells, or CAR-NK cells from other sources, at various ratios to match the target antigen expression has been raised and preliminarily tested. With the optimized CAR design and production protocols, a library of CAR-NK-92 cells targeting a wide variety of tumor antigens could be created to be applied as allogeneic off-the-shelf products in the future and to be mixed as personalized formulation for individual patients.

Regarding the CAR design, the costimulatory domains of CAR structure used in the clinical trials of CAR-NK therapy to date are T cell costimulatory molecules [4, 14]. We herein used CD28 and 4-1BB as costimulatory domains as they were reported to enhance the proliferation, survival, and cytotoxicity of CAR-T cells [15, 16]. For CAR gene transfer, lentiviral transduction was chosen because of a lower genotoxicity and insertional mutagenesis when compared to retroviral transduction [17]. The major advantage of CAR-NK cells over CAR-T cells is the capability to produce graft-versus-tumor effect without inducing GvHD in allogeneic setting even in HLA-mismatched recipients, the complication of which restricts the use of allogeneic CAR-T cells [4, 18]. Additionally, CAR-NK cells are potentially safer with respect to clinical implications as they induce a low incidence of cytokine storms and neurotoxicity [14, 19].

Majority of current clinical trials with CAR-NK cells use NK-92 cell line. Parental NK-92 cells were derived from NHL patient with severe chronic active EBV infection [20, 21], and thus, CAR-NK-92 cells need to be irradiated before clinical application to avoid the secondary tumorigenesis and potential EB virus susceptibility. In the first, small phase I clinical trials, the repeated infusion of *γ*-irradiated (10 Gy) CAR-NK-92 cells targeting CD33 at doses up to 5 × 10^9^ cells/patient was reported to be safe in three relapsed/refractory AML patients with no substantial adverse effects, although the clinical efficacy was not found to be significant [22], suggesting a good safety profile of these CAR-NK-92 cells similar to their parental NK-92 cells. Preclinical titration experiments demonstrated that a *γ*-irradiation at 10 Gy was sufficient to abrogate proliferation of CAR-NK-cells, without affecting their in vitro cytotoxicity for at least 48 h or reducing in vivo antitumor activity [23, 24]. It is worthy of note that the limited lifespan of CAR-NK-92 cells in vivo after irradiation could lower the risk of on-target, off-tumor toxicity.

Antigen escape from a downregulation or even loss of target antigen is one of the resistance mechanisms to CAR-T cell therapy [25]. Due to the antigen-unrestricted killing activity of NK cells, CAR-NK-92 cells can still be activated by their natural NK activating receptors even if the targeted antigens are lost. This CAR-independent mechanism of killing could also benefit to tumors with heterogeneous expression of the CAR target antigens. However, one of the drawbacks of CAR-NK-92 cells is the lack of CD16 (Fc*γ*RIII) expression [4]; thereby, they could not eliminate tumor cells through CD16-mediated antibody-dependent cell-mediated cytotoxicity (ADCC). Hence, CAR-NK-92 cells exert its tumor killing mainly through (i) granule-mediated cytotoxicity via perforin and granzymes; (ii) death receptor-mediated apoptosis, involving FasL and TRAIL; and (iii) the secretion of proinflammatory cytokines and chemokines, e.g., TNF-*α*, IFN-*Ɣ*, and CCL3 [4, 18, 26, 27].

Multitargeting approach is a potential strategy to expand the effectiveness of this novel immunotherapy, as it might lower the resistance and relapse rate of cancer. In MM, a decrease in CD138 expression has been observed during the course of clinical treatment in some patients and in clonogenic MM cells [28, 29], which are thought to be responsible for MM progression. A small subset of CD138-negative and CD19-positive MM cells are believed to be stem-like cells, also known as MM progenitor cells. It was reported that up to 4% of CD19-positive MM cells were detected in bone marrow aspirates of 103 MM patients [30]. CAR-NK-92 cells targeting CD19 and CD138 may exhibit improved clinical efficacy in relapsed and/or refractory MM. Notably, the coexpression of CD19 and CD138 might be detected in certain cases of LPL, B-CLL, ALL, and AML [8], as example in human ALL-derived REH cells herein, and hence, CAR-NK-92 cells targeting CD19 and CD138 may benefit those of hematologic malignancies as well.

Several multitargeting CAR-T cell therapies in clinical trials include (i) pooled CAR-T cells by a mixture of two different CAR-T cells targeting different antigens; (ii) dual CAR-T cells targeting two different antigens in a single CAR-T cells; (iii) tandem CAR-T cells, where two antigen binding domains are connected in tandem in a single CAR; and (iv) trivalent CAR-T cells targeting three different antigens in a single CAR-T cells [31]. However, there are not many studies on the multitargeting CAR-NK cells. With the feasibility for off-the-shelf manufacturing, we believe that a library of CAR-NK-92 cells or CAR-NK cells from other sources, including donor-derived peripheral blood mononuclear cells (PBMC), umbilical cord blood, and those derived from CD34-positive hematopoietic stem/progenitor cells (HSPCs) or induced pluripotent stem cells (iPSCs), could be created. For the concept of individual patients' optimized therapy, surface antigens of patient's tumors will be detected and spontaneous mixture of multiple CAR-NK cells at the right combinations will be formulated to match the antigen expression profiles. Additionally, the cytotoxicity of those CAR-NK formulations can be first evaluated in patient's primary cells in vitro in order to predict the best response. A sequential administration of different CAR-NK cells, termed cocktail immunotherapy, might be applied as in the case of CAR-T cells [32].

## 5. Conclusions

In summary, our findings reveal a promising CAR design and production protocol for single- and multiantigen-targeted CAR-NK-92 cells against various hematologic malignancies. The generated CAR-NK-92 cells targeting CD19 and/or CD138 antigens that employ CD28, 4-1BB, and CD3*ζ* signaling displayed high and selective cytotoxicity against established leukemia, lymphoma, and MM cells in vitro. Further studies should focus on the generation of the library of CAR-NK-92 cells targeting various novel tumor antigens, using current good manufacturing practice- (cGMP-) compliant transduction and expansion methodologies. This information represents the basis for further in vivo studies and future progress to clinical trials.

## Data Availability

The data used to support the findings of this study are included within the article.

## Supplementary Materials

Supplementary Materials Supplementary Table S1: efficacy of various CAR-NK-92 cells targeting CD19 or CD138 tumor antigen.
