# Supplementary material for: Selective Cytotoxicity of Single and Dual Anti-CD19 and Anti-CD138 Chimeric Antigen Receptor-Natural Killer Cells against Hematologic Malignancies
Source: J Immunol Res. 2021 Jul 11;2021:5562630. doi: 10.1155/2021/5562630 (PMC8289607; doi:10.1155/2021/5562630)
Supplement: Supplementary Materials — Supplementary Table S1: efficacy of various CAR-NK-92 cells targeting CD19 or CD138 tumor antigen. [file 5562630.f1.pdf]

Selective cytotoxicity of single and dual anti-CD19 and anti-CD138 chimeric antigen receptor-natural killer cells against hematologic malignancies

Sudjit Luanpitpong,[1*] Jirarat Poohadsuan,[1] Phatchanat Klaihmon,[1] Surapol Issaragrisil[1,2,3]

[1]Siriraj Center of Excellence for Stem Cell Research, Faculty of Medicine Siriraj Hospital, Mahidol University, Bangkok, Thailand

[2]Division of Hematology, Department of Medicine, Faculty of Medicine Siriraj Hospital, Mahidol University, Bangkok, Thailand

[3]Bangkok Hematology Center, Wattanosoth Hospital, BDMS Center of Excellence for Cancer, Bangkok, Thailand

Correspondence: Sudjit Luanpitpong, Siriraj Center of Excellence for Stem Cell Research, Faculty of Medicine Siriraj Hospital, Mahidol University, 2 Siriraj Hospital, Bangkoknoi, Bangkok 10700, Thailand; Tel.: +66 2 419 2907; Email: suidjit@gmail.com.

Supplementary information: Supplementary materials include Supplementary Table S1.

Supplementary Table S1. Efficacy of various CAR-NK-92 cells targeting CD19 or CD138 tumor antigen.

CAR design (generation)	Type of effector (E) NK-92 cells	Transduction / transfection efficiency	Type of target (T) cells	E:T ratio (incubati on time	Detection of cytotoxicity	Efficacy of CAR-NK-92 cells (vs control)	References
**CAR-NK-92 cells targeting CD19**							
scFv-CD28-4-1BB-CD3ζ (3rd gen)	NK-92	F(ab’)2 >80%	K562 (negative)	1:5 (4 h)	Annexin V+ (Annexin V/7-AAD)	25.24 ± 0.71% (vs 30.19 ± 3.31)	Current study
scFv-CD3ζ (1st gen)		Myc-tagged CAR N/A%		1:1 (4 h)	PI+	Approx. 15% (vs 10%)	[1]
scFv-CD3ζ (1st gen)		F(ab’)2 approx. 50%		5:1 (4 h)	PI+	61.7 ± 4.4% (vs 66.1 ± 0.1%)	[2]
scFv-CD28-4-1BB-CD3ζ (3rd gen)	NK-92	F(ab’)2 >80%	Raji (CD19)	1:5 (4 h)	Annexin V+ (Annexin V/7-AAD)	80.99 ± 4.14% (vs 64.12 ± 9.26)*	Current study
scFv-CD3ζ (1st gen)		F(ab’)2 >95%		2.5:1 (4 h)	PI+	Approx. 80% (vs 45%)	[3]
scFv-CD3ζ (1st gen)		scFv 95%		1:4 (24h)	Annexin V+ (Annexin V/7-AAD)	Approx. 45% (vs 15%)**	[4]
scFv-CD28 -CD3ζ (2nd gen)		Myc-tagged CAR N/A%		1:1 (2 h)	PI+	Approx. 60% (vs 10%)*	[5]
scFv-CD137- CD3ζ (2nd gen)				1:1 (6 h)		Approx. 45% (vs 20%)*	
scFv-CD28-4-1BB-CD3ζ (3rd gen)	NK-92	F(ab’)2 >80%	H929 (CD138)	1:5 (4 h)	Annexin V+ (Annexin V/7-AAD)	28.28 ± 4.17% (vs 36.30 ± 2.87)	Current study
scFv-CD28-4-1BB-CD3ζ (3rd gen)	NK-92	F(ab’)2 >80%	RPMI-8226 (CD138)	1:5 (4 h)	Annexin V+ (Annexin V/7-AAD)	39.9 ± 4.30% (vs 33.32 ± 6.92)	Current study

scFv-CD28-4-1BB-CD3ζ (3rd gen)	NK-92	F(ab')2 >80%	REH (CD19 and CD138)	1:5 (4 h)	Annexin V+ (Annexin V/7-AAD)	65.24 ± 2.69% (vs 5.83 ± 1.76)**	Current study
scFv-CD3ζ (1st gen)		F(ab')2 approx. 50%		5:1 (4 h)	PI+	76.3 ± 4.7% (vs 15%)**	[2]
scFv-CD3ζ (1st gen)		Myc-tagged CAR N/A%		1:1 (4 h)	PI+	Approx. 15% (vs 0%)**	[1]
**CAR-NK-92 cells targeting CD138**							
scFv-CD28-4-1BB-CD3ζ (3rd gen)	NK-92	F(ab')2 >80%	K562 (negative)	1:5 (4 h)	Annexin V+ (Annexin V/7-AAD)	32.25 ± 2.47% (vs 30.19 ± 3.31)	Current study
scFv-CD3ζ (1st gen)	NK-92MI	EGFP-tagged >95%		1:1 (4 h)	Annexin V+ (Annexin V/PI)	Approx. 60% (vs 55%)	[6]
scFv-CD28-4-1BB-CD3ζ (3rd gen)	NK-92	F(ab')2 >80%	Raji (CD19)	1:5 (4 h)	Annexin V+ (Annexin V/7-AAD)	63.20 ± 1.27% (vs 64.12 ± 9.26)	Current study
scFv-CD28-4-1BB-CD3ζ (3rd gen)	NK-92	F(ab')2 >80%	H929 (CD138)	1:5 (4 h)	Annexin V+ (Annexin V/7-AAD)	61.28 ± 0.05% (vs 36.30 ± 2.87)**	Current study
scFv-CD3ζ (1st gen)	NK-92MI	EGFP-tagged CAR >95%		1:1 (4 h)	Annexin V+ (Annexin V/PI)	Approx. 40% (vs 20%)**	[6]
scFv-CD28-4-1BB-CD3ζ (3rd gen)	NK-92	F(ab')2 >80%	RPMI-8226 (CD138)	1:5 (4 h)	Annexin V+ (Annexin V/7-AAD)	57.46 ± 1.88% (vs 33.32 ± 6.92)**	Current study
scFv-CD3ζ (1st gen)	NK-92MI	EGFP-tagged >95%		1:1 (4 h)	Annexin V+ (Annexin V/PI)	Approx. 30% (vs 15%)*	[6]
scFv-CD28- CD3ζ (2nd gen)		Fc 99.9%		1:4 (6 h)	7-AAD+	Approx. 45% (vs 25%)	[7]
scFv-CD28-4-1BB-CD3ζ (3rd gen)	NK-92	F(ab')2 >80%	REH (CD19 and CD138)	1:5 (4 h)	Annexin V+ (Annexin V/7-AAD)	18.45 ± 1.64% (vs 5.83 ± 1.76)**	Current study
**CAR-NK-92 cells targeting CD19 and CD138** – Data from other studies are not available.							

*p < 0.05, **p < 0.01 versus control in the same study. Abbreviation: PI, propidium iodide.

References

1. Romanski A, Uherek C, Bug G, Seifried E, Klingemann H, Wels WS, et al. CD19-CAR engineered NK-92 cells are sufficient to overcome NK cell resistance in B-cell malignancies. J Cell Mol Med. 2016 Jul;20(7):1287-1294.
2. Boissel L, Betancur M, Wels WS, Tuncer H, Klingemann H. Transfection with mRNA for CD19 specific chimeric antigen receptor restores NK cell mediated killing of CLL cells. Leuk Res. 2009;33(9):1255-1259.
3. Boissel L, Betancur M, Lu W, Wels WS, Marino T, Van Etten RA, et al. Comparison of mRNA and lentiviral based transfection of natural killer cells with chimeric antigen receptors recognizing lymphoid antigens. Leuk Lymphoma. 2012;53(5):958-965.
4. Ravi D, Sarkar S, Purvey S, Passero F, Beheshti A, Chen Y, et al. Interaction kinetics with transcriptomic and secretory responses of CD19-CAR natural killer-cell therapy in CD20 resistant non-hodgkin lymphoma. Leukemia. 2020;34(5):1291-1304.
5. Oelsner S, Friede ME, Zhang C, Wagner J, Badura S, Bader P, et al. Continuously expanding CAR NK-92 cells display selective cytotoxicity against B-cell leukemia and lymphoma. Cytotherapy. 2017;19(2):235-249.
6. Jiang H, Zhang W, Shang P, Zhang H, Fu W, Ye F, et al. Transfection of chimeric anti-CD138 gene enhances natural killer cell activation and killing of multiple myeloma cells. Mol Oncol. 2014;8(2):297-310.
7. Zhao S, Han Z, Ji C, An G, Meng H, Yang L. The research significance of concomitant use of CAR-CD138-NK and CAR-CD19-NK to target multiple myelomas. Eur J Inflamm. 2018;16:1-9. doi: 10.1177/2058739218788968.
